# Cortical sources of ERP in prosaccade and antisaccade eye movements using realistic source models

**DOI:** 10.3389/fnsys.2013.00027

**Published:** 2013-07-02

**Authors:** John E. Richards

**Affiliations:** Department of Psychology, Institute for Mind and Brain, University of South CarolinaColumbia, SC, USA

**Keywords:** prosaccades, antisaccades, eye movements, cortical source analysis, ERP

## Abstract

The cortical sources of event-related-potentials (ERP) using realistic source models were examined in a prosaccade and antisaccade procedure. College-age participants were presented with a preparatory interval and a target that indicated the direction of the eye movement that was to be made. In some blocks a cue was given in the peripheral location where the target was to be presented and in other blocks no cue was given. In Experiment 1 the prosaccade and antisaccade trials were presented randomly within a block; in Experiment 2 procedures were compared in which either prosaccade and antisaccade trials were mixed in the same block, or trials were presented in separate blocks with only one type of eye movement. There was a central negative slow wave occurring prior to the target, a slow positive wave over the parietal scalp prior to the saccade, and a parietal spike potential immediately prior to saccade onset. Cortical source analysis of these ERP components showed a common set of sources in the ventral anterior cingulate and orbital frontal gyrus for the presaccadic positive slow wave and the spike potential. In Experiment 2 the same cued- and non-cued blocks were used, but prosaccade and antisaccade trials were presented in separate blocks. This resulted in a smaller difference in reaction time between prosaccade and antisaccade trials. Unlike the first experiment, the central negative slow wave was larger on antisaccade than on prosaccade trials, and this effect on the ERP component had its cortical source primarily in the parietal and mid-central cortical areas contralateral to the direction of the eye movement. These results suggest that blocked prosaccade and antisaccade trials results in preparatory or set effects that decreases reaction time, eliminates some cueing effects, and is based on contralateral parietal-central brain areas.

## Introduction

The prosaccade and antisaccade procedure has been useful in the study of the brain control of eye movements. These eye movements are studied with the presentation of a target in one of two peripheral locations. An eye movement is made either to the target (“prosaccade”) or away from the target to the opposite location (“antisaccade”). This procedure has been used in a wide variety of studies to examine visual attention and eye movement control (Everling and Fischer, 1998; Munoz and Everling, 2004) and may be useful for examining neuropsychological status (i.e., schizophrenia, McDowell and Clementz, 2001; ADHD, Klein et al., 2003). Several studies with non-human animals have shown that areas of the frontal cortex, such as the frontal eye fields (FEF), supplementary eye fields (SEF), dorsolateral prefrontal cortex (DPC), and prefrontal cortex are involved in the generation of eye movements and differ for prosaccade and antisaccade eye movements. Neuroimaging studies using PET, block-design fMRI, and event-related fMRI have examined these eye movements in human participants and have found activity in several of these brain areas (see review by McDowell et al., 2008). The human neuroimaging studies lack the temporal resolution used in the non-human studies and therefore may not be able to examine the neural processes that are time-locked to these eye movements. Alternatively, studies have recorded scalp event-related-potentials (ERP) and show several types of presaccadic ERP activity related to prosaccade and antisaccade eye movements (Brickett et al., 1984; Evdokimidis et al., 1996; Everling et al., 1997, 1998). Two studies used cortical source analysis to determine the brain areas responsible for the generation of the ERP linked to these eye movements (Richards, 2003; McDowell et al., 2005). This paper describes a study of college-age participants' ERP activity for prosaccade and antisaccade eye movements. The cortical sources of the ERP activity were studied with realistic source models based on individual MRIs, and the effect of mixed-choice trials and blocked trials on ERP components was studied.

Many neuroimaging studies of prosaccade and antisaccade eye movements in humans have used PET or fMRI and blocked designs. The first studies in this area used PET imaging and a blocked design (Fox et al., 1985; O'Driscoll et al., 1995; Sweeney et al., 1996; Doricchi et al., 1997). Participants were given blocks of prosaccade trials, blocks of antisaccade trials, and perhaps trials with steady fixation, and subtraction techniques were used to identify brain areas more active in eye movements than fixation, or differential activity in prosaccade and antisaccade blocks. Similar studies have been done using fMRI (Connolly et al., 2000; Kimmig et al., 2001; Matsuda et al., 2006; Domagalik et al., 2012). Although the results from these neuroimaging studies are not entirely consistent, several areas of the frontal cortex (FEF, SEF, DPC, ventromedial or ventrolateral PFC) are more active in these eye movements than during fixation, or more active in antisaccade than prosaccade testing blocks. Other brain areas show such activation, such as the superior parietal cortex, intraparietal sulcus, and extrastriate occipital cortex (see McDowell et al., 2008).

Some studies used event-related fMRI in order to do mixed-choice trial presentations and to link the brain areas to specific components for these eye movements (Cornelissen et al., 2002; Curtis and D'Esposito, 2003, 2006; Desouza et al., 2003; Ford et al., 2005; Brown et al., 2007; Dyckman et al., 2007; Ettinger et al., 2008). It is possible with event-related fMRI to use a design in which prosaccade and antisaccade trials are randomly intermixed (mixed-choice trials design). In mixed-choice fMRI experiments, BOLD activation in the fMRI may be the same size for prosaccade and antisaccade trials (Cornelissen et al., 2002; Dyckman et al., 2007). For example, Dyckman et al. (2007) used event-related fMRI and had a antisaccade trial block, a prosaccade trial block, and a mixed antisaccade-trial/prosaccade trial block. They found several brain areas that were more active on the antisaccade trials in the single-type block than the prosaccade trials in the single-type block, but which were not more active on antisaccade trials than prosaccade trials in the mixed-choice trial block. This suggests that some of the additional activation during antisaccade blocks is due to preparatory set psychological processes. Event-related fMRI studies also have used designs to separate brain areas involved in preparatory eye movement planning and eye movement generation (Ford et al., 2005; Brown et al., 2007; Ettinger et al., 2008). For example, Ford et al. (2005) distinguished the early part of a preparatory period (first 6 of 10 s), the latter part of a preparatory period (last 4 of 10 s), and events following the target and saccade (events: 0.5 s; fMRI 5 s; see Figure 1 in Ford et al., 2005). They found that the FEF, SEF, areas in the prefrontal cortex (DPC; anterior cingulate cortex) and posterior cortex (intraparietal sulcus, parietal-occipital sulcus) have preparatory activity that distinguishes prosaccades and antisaccades. Brown et al. (2007) used a mixed-choice trials event-related fMRI design with trials on which a signal indicated a prosaccade or antisaccade was to be made, and on some trials the signal was followed by a response whereas other trials the response was not made. In this study they found the no-response trials showed more frontal and parietal brain activity on antisaccade cues than for prosaccade cues even when a response was not made. They suggested that the preparatory set effects occurring in response to the movement type stimulus elicits the larger brain responses for antisaccade trials. This type of event-related design might distinguish between preparatory events and the events surrounding the eye movements.

The study of the brain control of antisaccade and prosaccade eye movements has been aided by using ERP. The ERP activity provides better temporal resolution than PET or fMRI and may be especially important in distinguishing the brain areas controlling eye movements near the generation of the saccades. There is a slow negative ERP component before eye movements that begins up to 1 s prior to saccade onset and has its maximum value over the vertex. In blocked designs (e.g., Everling et al., 1997, 1998) this ERP component has a larger amplitude and more widespread scalp distribution for antisaccade eye movement blocks than for prosaccade eye movement blocks. In mixed-choice trial designs, if the warning stimulus in the preparatory interval is informative about the upcoming saccade and there is a response stimulus indicating the eye movement, this potential may be larger on antisaccade than on prosaccade trials (Klein et al., 2000; Richards, 2003; but cf. Mueller et al., 2009). But if the warning stimulus is uninformative with respect to the eye movement type, and the response stimulus is a target that indicates the eye movement type, this ERP component still occurs but there is no difference between the amplitude of this component on prosaccade and antisaccade trials (Evdokimidis et al., 1996; Richards, 2003). The close link of this component to the preparatory period and its time course (500–1000 ms before saccade onset) suggest it represents the preparatory activity in mixed-choice trial designs, or response set in blocked designs, similar to the preparatory BOLD activity occurring in event-related fMRI studies. There is a slow positive ERP component about 30–300 ms prior to saccade onset and which occurs over central and parietal areas (Everling et al., 1997; Richards, 2003). This positive component is more closely linked to the eye movement itself. There is a small positive ERP component occurring about 70 ms prior to the eye movement over frontal pole electrodes contralateral to the eye movement that is larger on antisaccade than on prosaccade eye movements (Richards, 2003). These latter components represent control processes closely tied to eye movement execution. The time course of these components imply that they represent brain activity that cannot be studied in detail with PET or fMRI-BOLD methods, even in event-related fMRI designs.

Two studies used cortical source analysis of the ERP components found in antisaccade and prosaccade eye movements (Richards, 2003; McDowell et al., 2005). Cortical source analysis (Scherg, 1990; Scherg and Picton, 1991; Scherg, 1992; Huizenga and Molenaar, 1994) is a technique for estimating the location and amplitude of cortical areas that general the EEG. McDowell et al. (2005) used a block design and measured EEG and MEG activity in response to the onset of the cue to move the eyes and activity preceding the eye movement. Activity in response to the imperative stimulus occurred primarily in posterior areas (cuneus, middle occipital gyrus). The activity preceding saccade onset occurred in FEF, SEF, and DPC and was larger on antisaccade than prosaccade trials. They were able to detail activity with ms resolution through the period immediately preceding saccade onset. Richards (2003) used a mixed-choice trial design with experiment events that distinguished preparatory activity, presaccadic activity, and activity in response to the target onset. The slow negative ERP component was associated with preparatory target activity had its cortical sources in Brodmann areas 6, 9, and 11 (near FEF, SEF, and DPC). The activity associated with this region did not differ on prosaccade and antisaccade trials. It was concluded that this area is related most closely to target preparatory activity and that the blocked-trials design may be necessary to show a prosaccade-antisaccade difference in this activity in the ERP (cf., Richards, 2003 and Everling et al., 1997; Evdokimidis et al., 1996 or McDowell et al., 2005). Richards (2003) found a small positive ERP component about 70 ms prior to eye movement, contralateral to the eye movement, and larger on antisaccade than prosaccade trials. This ERP component had cortical sources located in Brodmann areas 10 (frontal pole), 11 (orbital-frontal gyrus), and 8. The close link of this ERP component with the eye movement suggests brain areas closer to the frontal pole are closely tied to eye movement execution. Richards' findings suggest that the brain areas associated with eye movement preparatory activity (e.g., parietal, FEF, SEF, DPC) can be separated from brain areas associated with eye movement execution (e.g., frontal pole, orbital-frontal gyrus) with source analysis of ERP.

There were two aims of the current study. The first aim was to examine the brain areas involved in antisaccade and prosaccade eye movements using cortical source analysis of ERP with realistic models based on individual participant MRIs. The high temporal resolution of the ERP might allow the distinction between brain preparatory activities for eye movement in response to stimulus demand and brain activities related to saccade execution that occur immediately prior to saccades. The procedures for eliciting prosaccade and antisaccade eye movements may consist of preparation for the target occurrence, evaluation of the target, and saccade execution. These activities could be distinguished in the time domain of ERPs (e.g., <1 s preceding saccades) but not in fMRI. The current study used high-density EEG recording (128 channels) in a targeted procedure with a mixed-choice trials design in Experiment 1 (Evdokimidis et al., 1996; Klein et al., 2000; Richards, 2003). College-age participants were tested in a targeted procedure in which a cue signals a 2 s preparatory interval followed by a target that indicates the direction and type of eye movement and is the imperative signal for the eye movement. The cue during the preparatory interval acts as a warning stimulus and may induce preparatory brain activity, which would be expected to be larger on antisaccade than on prosaccade trials. The cortical source analysis used structural MRIs from individual participants to restrict the source solution to the gray matter of that participant. This allowed the source locations to be restricted to gray matter locations for that participant and defined specific anatomical areas tailored to the individuals' anatomical space rather than a generic brain or normalized Talairach space (Ha et al., 2003).

The second aim of the current study was to examine the effect of blocked and mixed-choice trial presentations on the ERP responses during antisaccade and prosaccade eye movements. Experiment 2 consisted of a design in which antisaccade trials and prosaccade trials were presented in separate blocks, or presented in the mixed-choice design of Experiment 1. One conclusion from fMRI studies comparing block- and mixed-choice designs is that blocked designs may result in preparatory set effects and lead to larger and more widespread activation of brain areas in antisaccade trials in those brain areas involved in eye movement preparation (Cornelissen et al., 2002; Ford et al., 2005; Brown et al., 2007; Dyckman et al., 2007). The different areas showing activation in the cortical source analysis studies of McDowell et al. (2005) and Richards (2003) may be due to the use of the block design in the former and the mixed-choice design in the latter. The comparison of the mixed-choice trials and blocked-trials may help distinguish the effects often studied in ERP tasks and the block-effects found in fMRI studies. Experiment 2 also used high-density EEG recording and cortical source analysis of the ERP using realistic source models based on individual participant MRIs.

## Experiment 1

### Method

#### Participants

The participants were nineteen adults (9 F). The participants ranged in age from 20 to 41 at time of testing (mean = 25.6, *SD* = 5.35) and consisted of undergraduate and graduate students. All participants were of normal intelligence and had no medical problems. The research was approved by the Institutional Review Board for the Use of Human Subjects and informed consent to participate in the study was obtained from each participant.

#### Apparatus and stimuli

**Figure 1:**
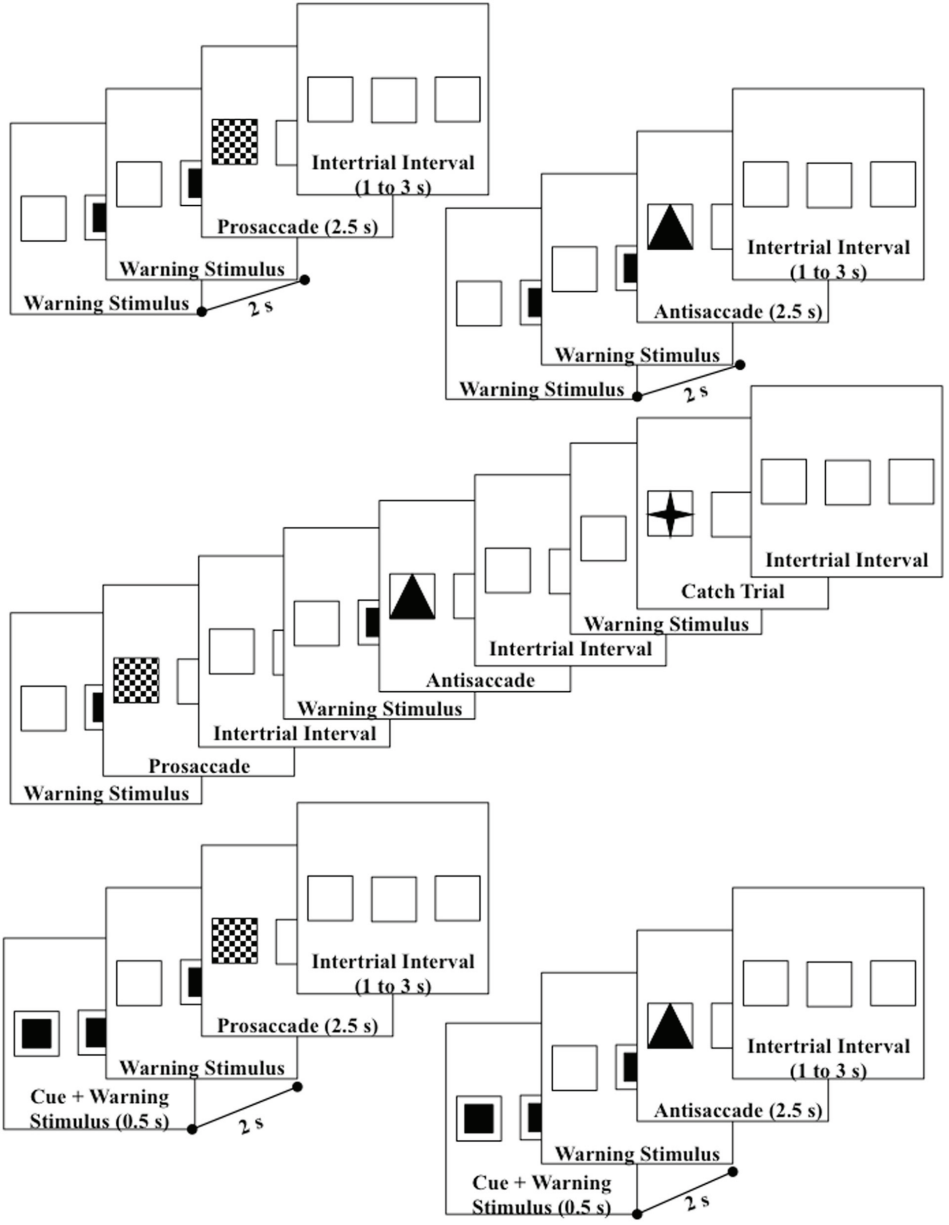
**Design of the trials—upper diagrams are uncued prosaccade and uncued antisaccade trials; middle diagram shows the uncued block presentations; lower diagrams are cued prosaccade and cued antisaccade**. Catch trials were also used which presented a small star in the target square. Both left target (shown) and right target (not shown) trials were used.

Each participant sat in a comfortable chair approximately 75 cm from a 29″ (56 × 42 mm) color video computer monitor (NEC Multisync XM29) displaying at 1280 horizontal and 1024 vertical pixels. The screen had a 2.6° square outline at each of three areas located in the center or 10° to the right or left of center which remained on at all times (Figure 1). The pretarget period was indicated by a small solid square in the center square outline. At target onset, the small solid square was removed, and a solid triangle, a checkerboard pattern, or a four-point star replaced one of the peripheral squares. The peripheral spatial cue consisted of a solid blinking square in the right or left target location.

#### Procedure

The participant sat in the chair and the viewing area facing the television monitor. The participant was informed that this was a study of the brain control of eye movements and was given instructions and practice in the procedure. Figure 1 shows the flow of each trial. The pretarget center square was presented for 2 s, followed by the presentation of the target for 2.5 s, followed by an interstimulus interval varying randomly from 1 to 3 s. The participants were instructed to make an eye movement toward the checkerboard target when it appeared (prosaccade), away from the triangle target to the opposite outline square (antisaccade), or to keep the eyes fixated in the center location when the four-point star appeared (catch trial). The targets were presented randomly and equally often on the left or right peripheral squares. The antisaccade, prosaccade, and catch trials were presented continuously, in random order for 5-trial blocks (2 antisaccade, 2 prosaccade, 1 catch trial). Figure 1 (middle panel) shows the continuous presentation sequence.

There were two cueing procedures used in the study. The “uncued” procedure consisted of the presentation of the pretarget and target stimuli without a cue (Figure 1, top two diagrams). The “cued” procedure consisted of the presentation of the blinking square for the first 500 ms of the pretarget period in the peripheral position where the target would occur (Figure 1, bottom two diagrams). Ten of the participants received these two conditions in alternating 5-min blocks, with as many presentations as possible within the blocks. The other 9 participants had multiple sessions, with the other sessions consisting of one of these two cueing conditions and some other conditions (see Richards, 2003 for conditions) but which were not analyzed in this study. There were 33 sessions used in the study.

#### Recording of EEG and segmenting of EEG for ERP

The EEG was recorded with a 128 channel EEG system (EGI, Inc.; Tucker, 1993; Tucker et al., 1994), referenced to vertex during recording and re-referenced algebraically to an average reference, recorded with 20 K amplification, at a sampling rate of 250 Hz, and with impedances below 100 kW^1^. The segments for the EEG were extracted for 1000 ms preceding target onset through the target onset until the saccade toward the target, and for 100 ms after saccade onset for target trials. The saccade to make the eye movement to the target was identified in the electrooculogram (EOG) recording (Matsuoka and Harato, 1983; Matsuoka and Ueda, 1986). Trials with incorrect eye movements or blinks were excluded from the analysis. For the ERP analysis the electrodes were grouped into sets of electrodes from the 128 channel GSN Sensornet that were close to the 10–20 locations into “virtual 10–20” electrodes (Table 1; see Supplementary Material). The ERP displays are based on these combined electrodes, and a multivariate approach to repeated measures was used for analysis by analyzing the groups of electrodes as multiple dependent variables and the experimental factors with a general linear models approach. The grouping of the electrodes and the multivariate analysis controlled for inflated error rates due to repeated tests and heterogeneity in the covariance matrix of the electrode effects.

**Table 1:** **Virtual 10–20 electrodes for GSN**.

FrontalZ	5, 12, 11, 16, 19, 10
CentralZ	7, 107, 32, 81, 55
ParietalZ	61, 68, 73, 79
OccipitalZ	72, 77, 76
FrontalPole1	22, 23, 18
FrontalPole2	14, 15, 9
Frontal3	25, 20, 21, 24
Frontal4	124, 119, 4, 3
Frontal7	34, 28, 35
Frontal8	122, 123, 117
Central3	30, 31, 36, 37, 42, 43
Central4	94, 104, 105, 106, 111, 112
Temporal3	40, 41, 46, 47
Temporal4	103, 109, 110, 116
Temporal5	51, 58, 59, 64, 65, 50
Temporal6	91, 92, 96, 97, 98, 102
Parietal3	52, 53, 54, 60
Parietal4	80, 86, 87, 93
Occipital1	66, 70, 71
Occipital2	85, 84, 90

#### Anatomical MRI, head segmenting, brodmann locations

A structural (anatomical) MRI was done for each participant in the study. Three of the MRIs were 3D T1-weighted images done on a 1.5T GE MRI, with 0.859 mm slices and 256 (axial) × 184 (sagittal) X 256 (coronal) resolution (Palmetto Imaging, Columbia, SC). The rest of the MRIs were 3D T1-weighted images done on a 3.0T Philips Intera MRI, rapid FLASH acquisition, 15.0° flip angle, *TE* = 5.7 ms, *TR* = 9.5 ms per FLASH line, effective *T*1 = 800 ms, 1.0 mm slices and 256 × 159 × 256 resolution (Center for Advanced Imaging Research, Medical University of South Carolina, Charleston, SC).

**Figure 2:**
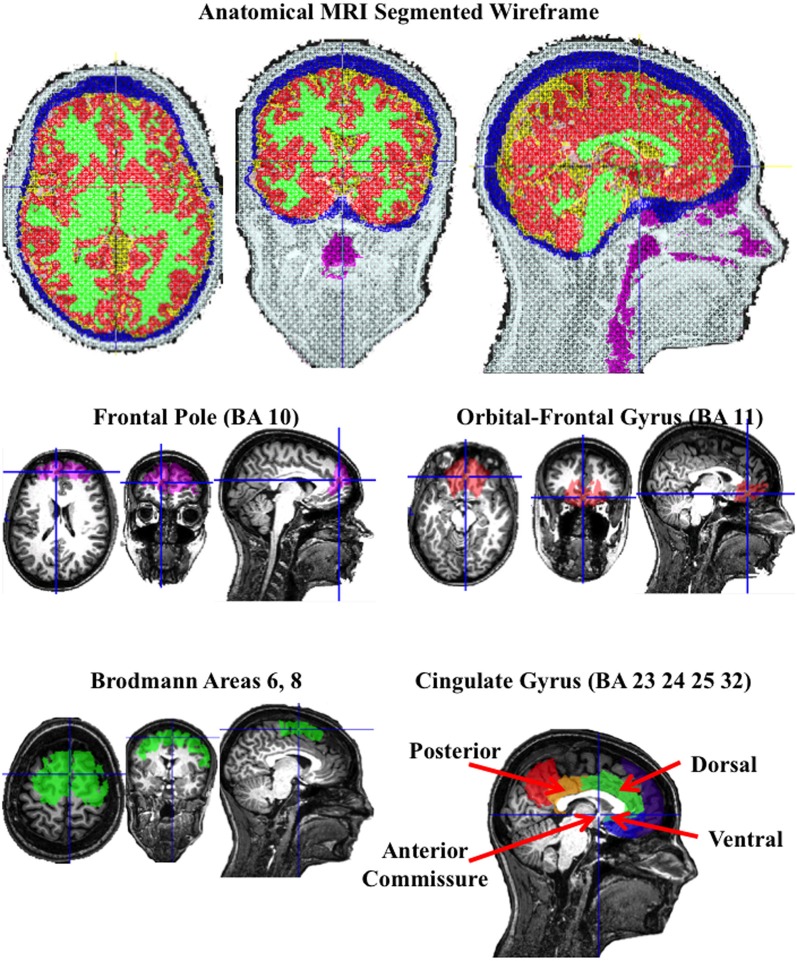
**A segmented wireframe from the anatomical MRI from one individual participant showing scalp (white), skull (blue), CSF (yellow), gray matter (red), white matter (green), and the nasal and throat air cavity (purple)**. The eye socket was also identified. The second row shows the “Region of Interest” (ROI) for the frontal pole and orbito-frontal gyrus, and the bottom row shows the Brodmann area 6 and 8 ROI and the cingulate gyrus ROIs.

The structural MRI of each participant was used to construct realistic head models for the cortical source analysis, so that the source analysis was based on a realistic model of that participants head. This included four steps (see Appendix). First, an average electrode placement map was generated for the participant. This was done by identifying fiducial electrode locations on the skull in the MRI volume, registering the fiducal locations on the skull to the same locations in the average electrode placement map, and transforming the electrode placement map to fit into the AC-coordinate system for that participant (Richards et al., unpublished). Second, the materials in the head were segmented, including scalp, skull, CSF, white matter, gray matter, nasal cavity, and eyes (Richards, 2005; Richards, unpublished). The segmenting resulted in a MRI volume with each voxel representing a specific material. Third, three-dimensional tetrahedral wireframes were computed that contained the location of each corner of the tetrahedron and the type of material making up the tetrahedron, using the MR Viewer module of the EMSE computer program (Source Signal, Inc.). Figure 2 (top row) shows the segmented wireframe from an anatomical MRI from one participant. Fourth, individual participant MRIs had anatomical areas defined by an atlas for that individual. The atlas either came from an average MRI template of 20–24 year old adults (Sanchez et al., 2012) that was registered/transformed to the individual participant's head space, or from atlases computed on the individual participants (Phillips et al., unpublished). The atlases were used to define several anatomical areas by identifying common designations from each of the atlases for the ROIs for that participant. There were nine ROIs chosen for which bilateral activity was represented by separate left and right regions, and six ROIs chosen along the midline for which bilateral activity was combined (Table 2 for midline regions; Table 3 for bilateral regions).

**Table 2:** **Regions-of-Interest for the individual participant atlases for central (non-lateralized) areas**.

Frontal pole (BA 10)
Harvard-Oxford atlas, area 1
Orbito-frontal gyrus (BA 11)
Hammers atlas, straight gyrus (right and left), areas 52, 53
Hammers atlas, medial orbital gyrus (right and left), areas 68, 69
Harvard-Oxford atlas, area 25
LPBA atlas, middle orbitofrontal gyrus (right and left), areas 29, 30
LPBA atlas, gyrus rectus (right and left), areas 33, 34
Ventral anterior cingulate, including subcallosal cortex
Hammers atlas, Subgenual anterior cingulate gyrus (right, left), areas 76, 77
Hammers atlas, Subcallosal area (right, left), areas 78, 79
Hammers atlas, Pre-subgenual anterior cingulate (right, left), areas 80, 81
Harvard-Oxford atlas, Subcallosal cortex (area 25)
Harvard-Oxford atlas, Paracingulate gyrus, area 28, below anterior commissure
Harvard-Oxford atlas, Cingulate gyrus, anterior division, area 29, below AC
Dorsal anterior cingulate, including paracingulate gryus
All areas were masked to be superior and anterior to the anterior commissure
Hammers atlas, Cingulate gyrus, anterior (supragenual) (right, left), areas 24, 25
Harvard-Oxford atlas, Paracingulate gyrus, area 28
Harvard-Oxford atlas, Cingulate gyrus, anterior division, areas 29
Posterior cingulate gyrus (Masked posterior to the anterior commissure)
Hammers atlas, Cingulate gyrus, posterior part (right, left), areas 26, 27
Harvard-Oxford atlas, Cingulate gyrus, posterior division, areas 30, 30
Superior parietal lobe
Hammers atlas, Superior parietal gyrus (right, left), atlas 62, 63
Harvard-Oxford atlas, Superior parietal lobule, area 18
LPBA atlas, Superior parietal gyrus (right, left), areas 43, 44

Phillips et al., unpublished, has list of all atlases and segmented areas.

**Table 3:** **Regions-of-Interest for the individual participant atlases for lateralized areas (separate left, right ROIs)**.

Frontal pole (BA 10)
Harvard-Oxford atlas, area 1, right and left mask from participant
Brodmann areas 6 and 8
Brodmann atlas, areas 6 and 8, right and left mask from participant
Brodmann areas 9 and 46, dorsolateral PFC
Brodmann atlas, areas 9 and 46, right and left mask from participant
Supramarginal gyrus
LPBA atlas, Supramarginal gyrus (right, left), areas 45, 46
Angular gyrus
LPBA atlas, Angular gyrus (right, left), areas 47, 48
Intraparietal sulcus
(1) Defined inferior parietal lobe, consisting of supramarginal gyrus and angular gyrus
LPBA atlas, Supramarginal gyrus (right, left), areas 45, 46
LPBA atlas, Angular gyrus (right, left), areas 47, 48
Hammers atlas, Remainder of parietal lobe (including supramarginal and angular gyrus; right, left), areas 32, 33
(2) Dilated the IPL by 3 mm
(3) Defined the superior parietal lobe
Hammers atlas, Superior parietal gyrus (right, left), atlas 62, 63
LPBA atlas, Superior parietal gyrus (right, left), areas 43, 44
(4) Dilated the SPL by 3 mm
(5) Found overlap of IPL and SPL 3 mm
Superior parietal lobe
Hammers atlas, Superior parietal gyrus (right, left), atlas 62, 63
Harvard-Oxford atlas, Superior parietal lobule, area 18
LPBA atlas, Superior parietal gyrus (right, left), areas 43, 44
Pre-central and post-central gyri
Hammers atlas, Precentral gyrus, Postcentral gyrus (right, left), atlas 50, 51, 60, 61
Harvard-Oxford atlas, Precentral gyrus, area 7
LPBA atlas, Precentral gyrus, postcentral gyrus (right, left), areas 27, 28, 41, 42
Residual frontal and temporal. Any area in manual lobar atlas in frontal or temporal lobes which were not included in the other ROIs

Phillips et al., unpublished, has list of all atlases and segmented areas.

**Figure 3:**
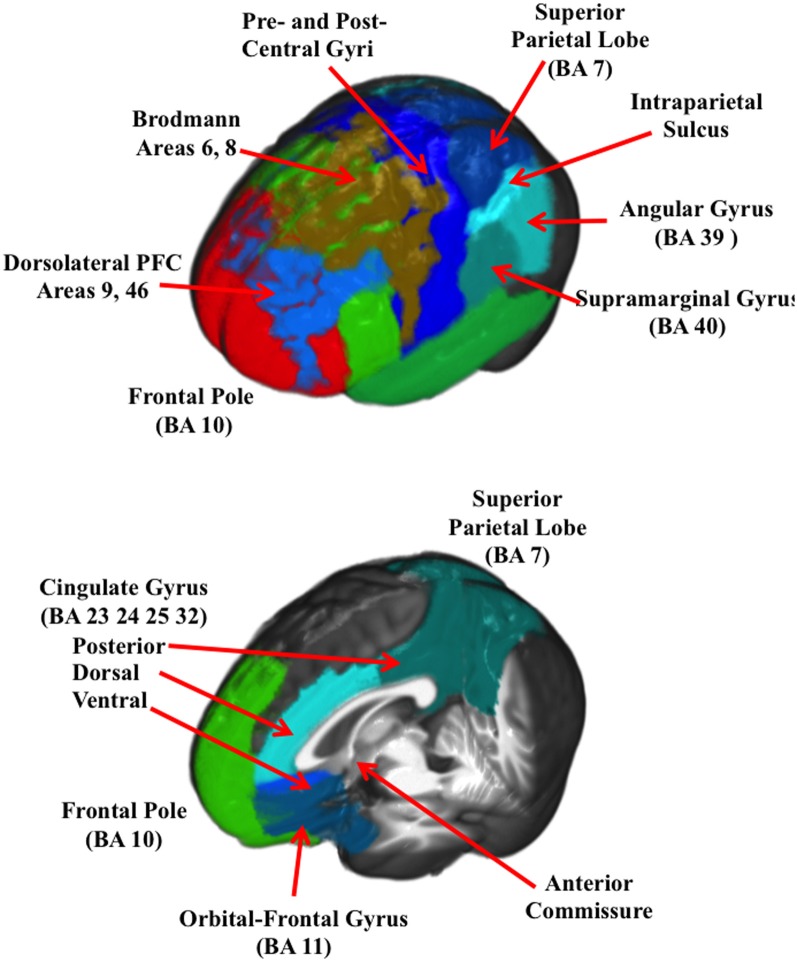
**A 3-D rendered brain showing the ROIs for the lateral regions (top figure) and the ROIs with only central regios (bottom figure)**.

Figure 2 (bottom two rows) show the ROIs for one individual for the frontal pole (BA 10), orbital-frontal gyrus (BA 11), and the cingulate cortex (areas 23, 24, 32, and 25). The cingulate cortex was further divided into three regions: posterior to the anterior commisure (posterior cingulate cortex), the dorsal area anterior and superior to the anterior commisure (dorsal anterior cingulate cortex), and the ventral portion of the anterior cingulate (ventral anterior cingulate cortex). Figure 3 shows the ROI source regions on a 3-D rendered brain.

#### Realistic cortical source analysis

Cortical sources estimated as the current source density of cortical source locations with current density reconstruction (CDR; Darvas et al., 2001) using sLORETA (Pascual-Marqui et al., 1994; Pascual-Marqui, 2002) as the constraint for the CDR. The electrode locations, source locations, and head model were used with EMSE's Data Analysis (Source Signal, Inc.) to estimate the forward model, inverse model, and current density reconstruction. The realistic cortical source models used a “finite-element method” (FEM) mapping of the electrical conductivity of the head to calculate the forward model. The FEM forward model was calculated offline with the Data Analysis module of the EMSE computer program (Source Signal, Inc.). The forward model and the ERP from the pretarget period were used to estimate a lead-field matrix representing the inverse model using the sLORETA restriction algorithm. This required the estimation of a lead-field matrix based on the realistic locations of the electrodes on the scalp, the source locations defined by the segmented gray matter and the location of the eyes, and the FEM model for the individual participant.

The pretarget or presaccadic ERP were used in a 4-ms by 4-ms segment averaged over the appropriate experimental factors and conditions, and the entire ERP segment was used to estimate the CDR for each ERP slice. This resulted in a MRI volume representing the source volumes at each sampled ERP time. The MRI volumes contain the CDR for each voxel in the source locations. The CDR were summed over each voxel of the ROI and divided by the total volume of the ROI. This results in an average current per mm value for each ROI. The ROIs for the analysis were anatomical areas determined on theoretical grounds or by reference to past cortical source analysis studies (Richards, 2003; McDowell et al., 2005) and PET or MRI neuroimaging studies (see Supplementary Material).

### Results

#### Saccade error and latency

The onset of the saccade from the center to the targeted square was analyzed. There were 8082 eye movements in the experiment, distributed approximately equally for antisaccades and prosaccades (4029 and 4053 eye movements, respectively) and across the experimental conditions (from 1952 to 2101 eye movements for each block-type/eye movement type combination). The error rate for the uncued and cued trials was approximately equal (3.24 and 3.29%, respectively), but there were slightly more errors on the antisaccade than on the prosaccade trials (2.50 and 4.04%, respectively), but errors on the two trial types (uncued, cued) were similar for prosaccade and antisaccade trials. The latency of the saccade onset from the target onset was analyzed by a repeated measures ANOVA^2^ with trial type (cued, uncued) and eye movement type (prosaccade, antisaccade) as factors. There were main effects for trial type, *F*_(1, 29)_ = 28.66, *p* < 0.001, movement type, *F*_(1, 29)_ = 86.09, *p* < 0.001, and an interaction between them, *F*_(1, 29)_ = 12.46, *p* < 0.001. As expected, saccades were faster on cued than uncued trials (*M* = 435.6, *N* = 4029, *SE* = 2.53; *M* = 473.0, *N* = 4053, *SE* = 2.38) and for prosaccade eye movements than antisaccade eye movements (*M* = 422.8, *N* = 4162, *SE* = 2.33; *M* = 487.9, *N* = 3920, *SE* = 2.51). The interaction between trial type and eye movement type occurred because the cue facilitated the reaction time of the prosaccade eye movements by 29 ms whereas it facilitated the antisaccade eye movements by 54 ms.

#### Grand average ERP

**Figure 4:**
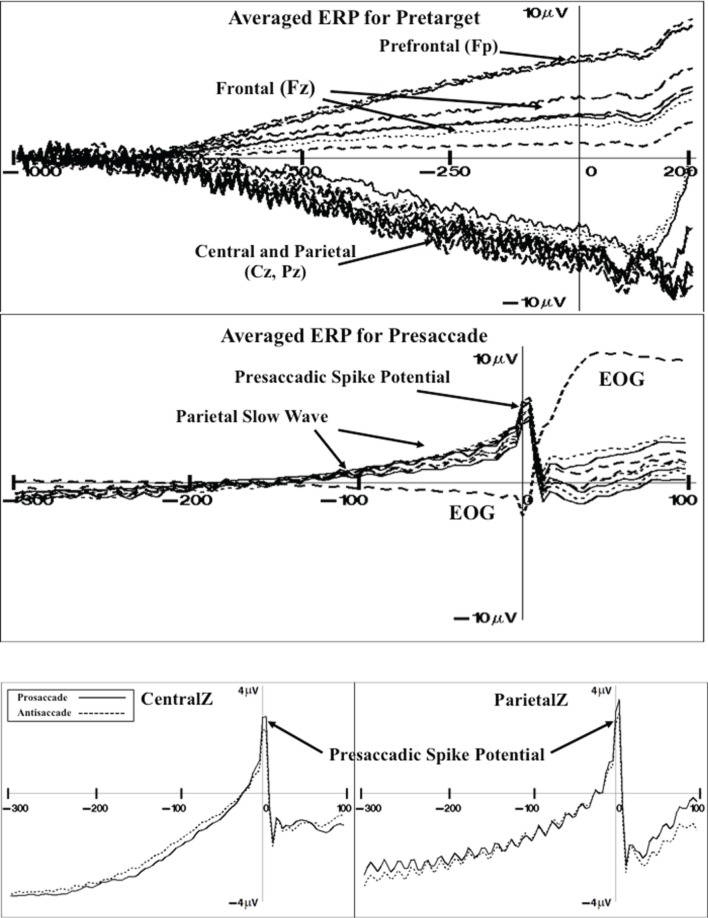
**Grand average ERP for pretarget activity for representative electrodes (top panel)**. The pretarget period shows frontal pole (Fp) and frontal (Fz) electrodes showing a slow positive ERP component simultaneous with central (Cz) and parietal (Pz) electrodes showing a slow negative ERP component (CNV). Grand average ERP for presaccade activity for representative electrodes **(middle panel)**. The presaccade activity shows the parietal slow ERP component and spike potential in the parietal leads. Grand average ERP for the presaccadic spike potential on the CentralZ and ParietalZ virtual electrode group for prosaccade and antisaccade eye movements **(bottom panel)**.

There were three ERP components that were examined as a function of the experimental variables. First, grand average ERP and topographical maps were constructed to display the target-locked ERP changes occurring in this task (e.g., Richards, 2003). Figure 4 (top panel) shows the pretarget ERP from 1 s before target onset through 200 ms of target onset for several frontal-central electrodes (baseline is 1.1–1.0 s before target onset). The pretarget ERP averages showed a positive slow component with maximal amplitude in the prefrontal scalp leads (e.g., FP1, FPz, FP2) that tapered off in the frontal scalp leads (Fz). There was a negative slow ERP component primarily in the central and parietal leads. The negative slow wave that occurred over the central areas of the scalp was analyzed with a multivariate approach to testing for the FrontalZ, CentralZ, ParietalZ, and OccipitalZ virtual electrode groups. The extent of the pretarget slow wave component was quantified by computing the mean ERP level in the last 50 ms of the pretarget interval, which is the target onset, minus the mean ERP level in the first 50 ms of the pretarget interval. This difference was analyzed with a Cue Type (2: uncued, cued) × Movement Type (3: prosaccade, antisaccade, catch) MANOVA. The only significant effect was a Cue Type main effect on the CentralZ virtual electrodes, Wilk's Λ = 0.5676, *F*_(6, 23)_ = 2.92, *p* = 0.0288. The negative slow wave was larger for the uncued trials than for the cued trials (*M*'s of CentralZ virtual electrode group = −5.65 and −5.00 μV, respectively). Since the participant did not know the type of eye movement in advance of the target, this indicates that the negative contingency was attenuated as a result of the spatial cue.

Figure 4 shows the presaccade ERP for several parietal electrodes (middle panel). This shows a slow positive component beginning about 150 ms prior to saccade onset (difference from −220 to −200 ms presaccade). The positive presaccadic potential shown in Figure 4 occurring immediately before saccade onset appears to be the “spike potential.”

The slow positive slow wave occurring primarily over the parietal areas was examined by computing the mean value in the presaccadic interval from about –50 to –20 ms preceding the saccade (Figure 4). This was analyzed for the FrontalZ, CentralZ, ParietalZ, and OccipitalZ electrode groups with a Cue Type (2) × Movement Type (2: prosaccade, antisaccade) for the trials on which a correct eye movement occurred. The only significant effect was a Movement Type main effect on the ParietalZ virtual electrodes, Wilk's Λ = 0.6131, *F*_(5, 25)_ = 3.15, *p* = 0.0242. The parietal slow wave was larger for the antisaccade trials than for the prosaccade trials (*M*'s = 2.33 and 1.89 μV, respectively). This slow wave was larger over the parietal leads contralateral to the eye movement. The side of the ERP data were switched so that the side of the eye movement was toward the right on each trial, and the contralateral parietal virtual electrodes (i.e., Parietal3) and ipsilateral electrodes (i.e., Parietal4) were analyzed with a Cue Type × Movement Type MANOVA. The ERP for the contralateral parietal electrode group was significantly affected by Movement Type, Wilk's Λ = 0.4935, *F*_(4, 26)_ = 6.67, *p* = 0.0008, with the parietal slow wave was larger before antisaccade eye movements than prosaccade eye movements.

The presaccadic spike potential was analyzed. The difference between the ERP in the intervals immediately preceding the saccade (−24 to −16 ms) and the ERP occurring at the time of the saccade (−8 through + 8 ms) was analyzed for the central electrode groups with a Cue Type (2) × Movement Type (2) MANOVA. The Movement Type factor affected both the CentralZ electrode group, Wilk's Λ = 0.4762, *F*_(6, 24)_ = 4.40, *p* = 0.0039, and the ParietalZ electrode group, Wilk's Λ = 0.5083, *F*_(5, 25)_ = 4.84, *p* = 0.0031. The presaccadic ms-by-ms changes in the ERP of the CentralZ and ParietalZ are shown in Figure 4. These figures used the intervals immediately preceding the saccade as the baseline. The presaccadic spike potential was larger on trials on which a prosaccade eye movement occurred than on trials on which an antisaccade eye movement occurred. The difference in this spike potential between eye movement types also occurred over lateral parietal leads (Parietal3, Parietal4) but not over central or occipital center or lateral electrodes (no effects for CentralZ, OccipitalZ, Central3, Central4, Occipital1, Occipital2).

#### ERP source analysis

**Figure 5:**
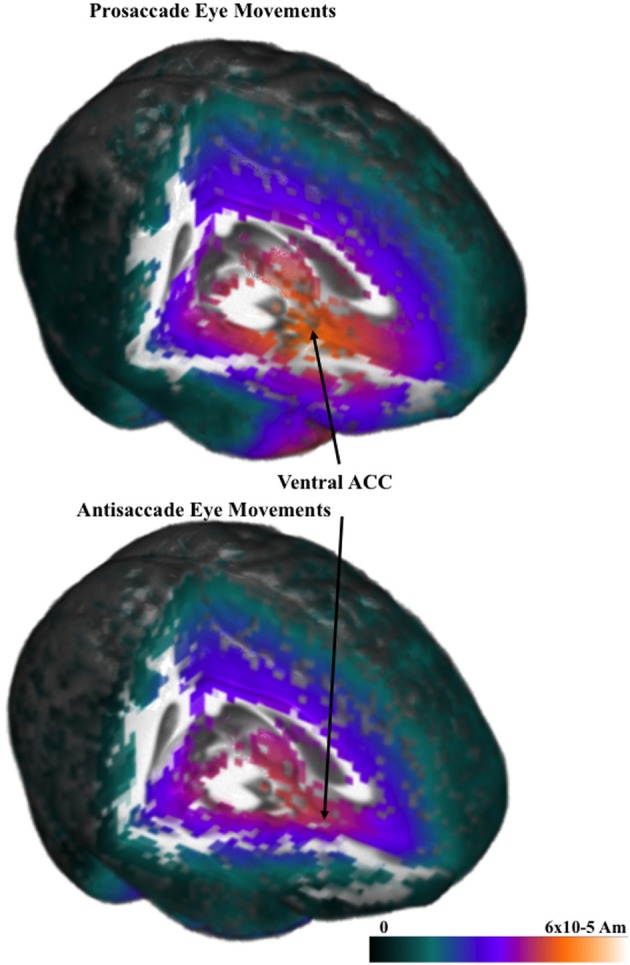
**Current density reconstruction for the pressaccadic spike potential for prosaccade and antisaccade eye movements**.

**Figure 6:**
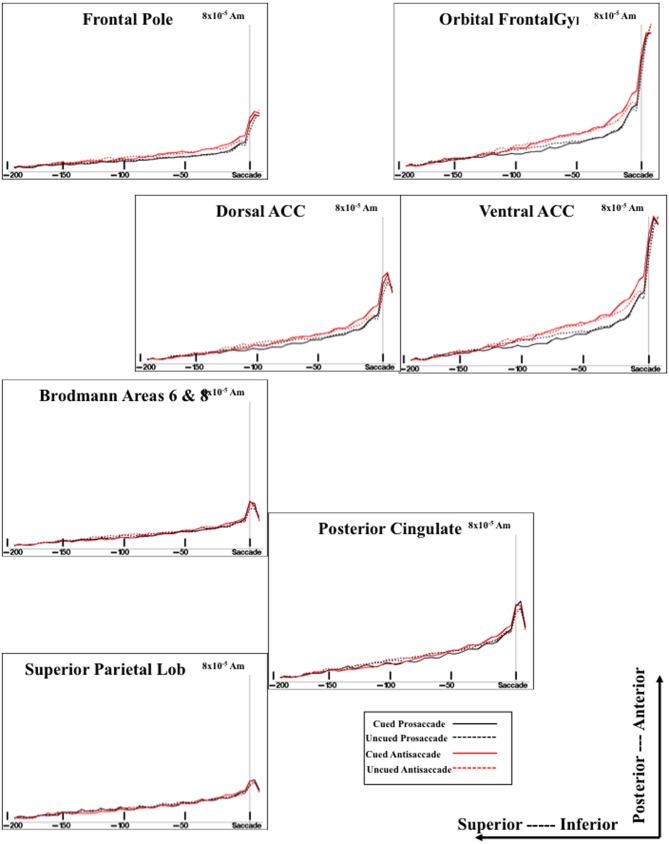
**Temporal unfolding of the current density sources for the presaccadic ERP activity from selected ROI areas**. This graph shows both the difference between cued and uncued trials on the presaccadic spike potential, and between the antisaccade and prosaccade trials on the presaccadic parietal slow wave.

The cortical sources of the ERP were analyzed. I restricted these analyses to examine the presaccadic spike potential and the presaccadic positive slow wave, which were found in the ERP analyses to be significantly affected by the type of eye movement. Figure 5 shows a 3-D rendering of the current density reconstruction of the ERP occurring at the peak of the spike potential, separately for prosaccades and antisaccades. The primary area showing activity was below the anterior cingulate in the ventral anterior cingulate and the orbital-frontal gyrus. This activity appears to be greater for prosaccade (top panel) than for antisaccade (bottom panel) eye movements. Figure 6 shows the ms-by-ms mean *nAm* for selected ROIs. The ventral anterior cingulate and the orbital frontal gyrus both showed the largest increase in the positive slow wave and a large spike potential.

The sources of the presaccadic positive slow wave were examined by computing a mean current density value in the interval from about −50 to −20 ms preceding the saccade. This value was analyzed with a ROI (e.g., frontal pole, orbital frontal gyrus, Brodmann areas 6 and 8, dorsal ACC, ventral ACC, pre- and post-central gryi, superior parietal lobe, posterior cingulate, intraparietal sulcus, supramarginal gyrus, angular gyrus, dorsolateral PFC) × Cue Type (2) × Movement Type (2) ANOVA. The positive slow wave current density was significantly affected by the ROI, *F*_(15, 375)_ = 34.33, *p* < 0.0001, and an interaction between ROI and eye movement type, *F*_(15, 255)_ = 3.32, *p* < 0.0001. The interaction reflected a significant effect of movement type for the current density coming from the ventral ACC and orbital frontal gyrus. The current density was larger for antisaccades than for prosaccades (Figure 6, for ventral ACC and orbital frontal gyrus). There were smaller (non-significant) effects for the Brodmann areas 6 and 8, and the dorsolateral PFC in the same direction.

The cortical sources of the presaccadic spike potential were examined by calculating the difference for the current density from the period immediately preceding the saccade [immediately preceding the saccade (−24 to −16 ms) and the ERP occurring at the time of the saccade (−8 through + 8 ms)]. This was analyzed with a ROI × Cue Type × Movement Type ANOVA. There were main effects of the cue type, *F*_(1, 17)_ = 17.78, *p* = 0.0126, ROI type, *F*_(15, 375)_ = 57.83, *p* < 0.0001, and an interaction of cue type and ROI type, *F*_(15, 255)_ = 7.41, *p* < 0.0001. As with the positive slow wave, the cue type effects occurred in the ventral ACC and the orbital frontal cortex, and to a lesser degree, in the dorsal ACC.

### Discussion

There were three types of ERP activity found in this study that replicated findings from other studies. First, there was a negative potential shift in the ERP that occurred before target onset and was associated with the preparatory interval of the task rather than the saccade itself. Several studies of ERP activity in the prosaccade and antisaccade task report this negative shift in the EEG that begins up to 1 s prior to saccade onset and has its maximum over the vertex (Brickett et al., 1984; Evdokimidis et al., 1996; Everling et al., 1997, 1998; Klein et al., 2000; Richards, 2003; Mueller et al., 2009). Two of the ERP components were closely tied to events surrounding the saccade itself. The second type of ERP activity was the slow positive potential shift in ERP beginning about 100 ms before saccade onset with maximum values over central and parietal leads. This positive slow component over parietal leads is not unique to studies of the antisaccade and prosaccade but is also found in voluntary eye movements. Some studies report no difference in this component between antisaccade and prosaccade trials (Evdokimidis et al., 1996; Everling et al., 1997, 1998; Richards, 2003), but in the current study it was larger for antisaccade than for prosaccade eye movements The third type of ERP component was the sharp spike in ERP over the central and parietal leads called the “spike potential.” In one study this potential was larger for antisaccade trials than prosaccade trials (blocked design, Everling et al., 1997) but generally this potential was the same on prosaccade and antisaccade trials (Evdokimidis et al., 1996; Everling et al., 1997, 1998; Klein et al., 2000; Richards, 2003). In the current study it was larger on the prosaccade eye movement trials than on the antisaccade eye movements trials.

The cortical sources of the presaccadic eye movements were examined with current density reconstruction using realistic head models. The sources for the components around the presaccadic eye movements both were found primarily in the ventral portion of the anterior cingulate cortex and the orbital frontal gyrus. The current density was larger for the antisaccade eye movements than for prosaccade eye movements in the period similar to the positive slow wave in the parietal scalp leads. This difference, and the timing of the current density over the presaccadic interval (e.g., Figure 6) was similar to that of the presaccadic positive slow wave (Figure 4). These findings suggest that this area is the cortical source generating this ERP component on the scalp. The spike potential in the ERP also appears to be localized to the same cortical area. At least, the timing of the spike potential occurring in the ERP was similar to what was found in the ms-by-ms current density reconstruction values in these ROIs. It is interesting that the spatial cue affects the current density in this ROI, whereas the type of eye movement affected the ERP component.

The second experiment was designed to test the effects of presenting stimuli in a mixed-choice design to presenting trials in a blocked design. Studies fMRI and ERP have shown brain activity during antisaccades that is larger than prosaccades in blocked trials, and many of these differences disappear for mixed-choice trials (e.g., Dyckman et al., 2007). Similarly, event-related fMRI studies that separate preparatory periods and execution periods find many more areas in which the preparatory brain activity is larger for antisaccade than for prosaccade eye movements. This suggests that the blocked trials result in preparatory psychological processes that do not exist in mixed-choice trials. One advantage of the timing resolution of ERP and the instantaneous response of the electrical changes in the brain is that eye movement preparatory and execution activity in the brain might be distinguished in either blocked or mixed-choice designs. The second experiment was designed to compare the mixed-choice trial design with a design in which prosaccade or antisaccade trials were presented in separate blocks.

## Experiment 2

### Method

#### Participants

The participants were eleven adults (7F). The participants ranged in age from 19 to 34 at time of testing (mean = 25.4, *SD* = 4.84) and consisted of undergraduate and graduate students.

#### Procedure

**Figure 7:**
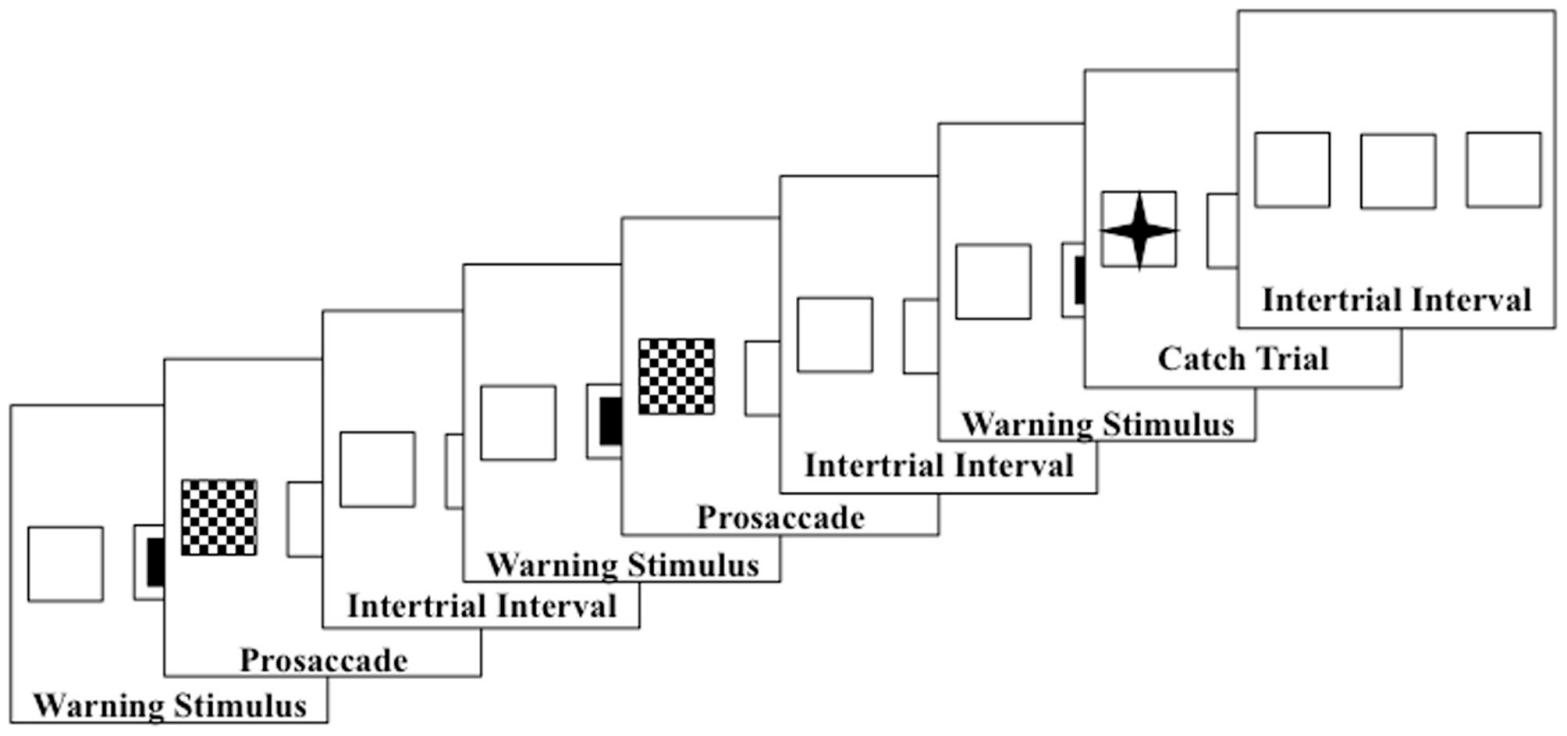
**An example blocked trials presentation sequence**. The sequence shows the blocks of uncued prosaccade trials.

The procedure differed from Experiment 1. There were six types of presentations. Four presentations were blocked according the type of eye movement and cueing procedure. This resulted in four blocked conditions: (1) uncued prosaccade trial blocks, (2) uncued antisaccade trial blocks, (3) cued prosaccade trial blocks, and (4) cued antisaccade blocks. Two additional mixed-choice trial blocks were given: (5) uncued prosaccade/antisaccade mixed trial blocks, (6) cued prosaccade mixed trial blocks. Figure 7 shows a representative set of trials for the uncued prosaccade trial block; Figure 1 (middle panel) shows the corresponding uncued mixed-choice prosaccade and antisaccade trial blocks. All trial blocks included catch trials, and trials were presented in random order for 5-trial sequences (4 prosaccade and 1 catch trial for prosaccade blocks; 4 antisaccade and 1 catch trial for antisaccade blocks; 2 antisaccade, 2 prosaccade, 1 catch trial for mixed-choice trial blocks). The six presentations types resulted in prosaccade and antisaccade trial data for cued- and non-cued presentations, and for blocked and mixed-choice presentations.

Each participant received all six types of blocked presentation, in 5-min blocks, with the order of presentation being randomly chosen without replacement for the six block types.

#### Other methods

All the MRIs for Experiment 2 were 3D T1-weighted images done on a 3.0T Philips Intera MRI, with 1.0 mm slices and 256 × 159 × 256 resolution (Center for Advanced Imaging Research, Medical University of South Carolina, Charleston, SC).

### Results

#### Saccade error and latency

**Figure 8:**
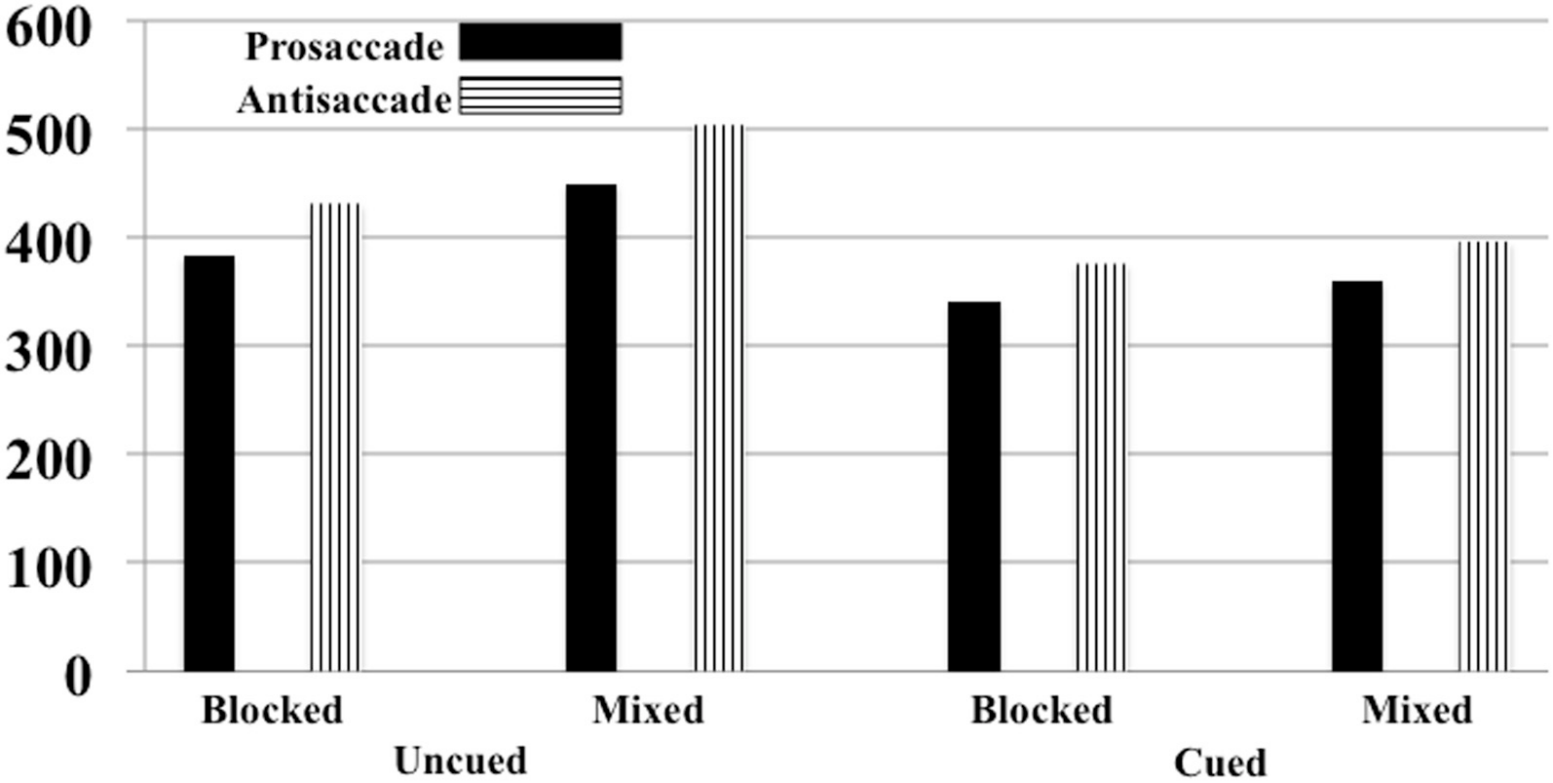
**Reaction time for moving the eyes from the center to the target for Experiment 2**. This is presented separately for cued and uncued trials, blocked and mixed-choice procedure, and prosaccade and antisaccade eye movements. The SE of the means for the six conditions ranged from 4.44 to 5.90.

The onset of the saccade from the center to the targeted square was analyzed. There were 4436 eye movements in the experiment, distributed approximately equally for antisaccades and prosaccades (2100 and 2166 eye movements, respectively) and across the experimental conditions (from 512 to 580 eye movements for each block-type/eye movement type combination). The error rate for the uncued and cued trials was approximately equal (2.54 and 2.14%, respectively), as were errors on the antisaccade than on the prosaccade trials (2.22 and 2.24%, respectively), and slightly more errors on mixed-choice trials than on blocked trials (2.80 and 1.91%, respectively). The latency of the saccade onset from the target onset was analyzed by a repeated measures ANOVA with trial type (cued, uncued), eye movement type (prosaccade, antisaccade), and procedure type (blocked, mixed) as factors. There were main effects for trial type, *F*_(1, 10)_ = 112.28, *p* < 0.001, movement type, *F*_(1, 10)_ = 20.74, *p* = 0.0011, procedure type, *F*_(1, 10)_ = 108.28, *p* < 0.001, and an interaction between trial type and procedure type, *F*_(1, 10)_ = 36.39, *p* < 0.001. Figure 8 shows the RT's as a function of the three experimental facts. As found in Experiment 1, saccades were faster on cued than uncued trials, and faster for prosaccades than antisaccades. The trial type by procedure type was due to a larger cueing effect on mixed-choice trials (98 ms) than on blocked trials (48 ms).

I examined the Trial Type × Movement Type effect separately for the blocked and mixed-choice trials. For the mixed-choice trials, similar to Experiment 1, there was a significant interaction between trial type and movement type for the mixed-choice trials (*p* = 0.0530). However, the interaction of trial type and movement type was not significant on the blocked trials done in Experiment 2 (*p* = 0.2177). For the mixed-choice trials, similar to Experiment 1, the cue facilitated the reaction time of the prosaccade eye movements by 41 ms whereas it facilitated the antisaccade eye movements by 107 ms. Alternatively, on blocked trials used in the current experiment, the cue effect was similar for prosaccade (43 ms) and antisaccade (53) trials, and was approximately the same size as the prosaccade cueing condition on the mixed-choice trials in Experiment's 1 and 2.

#### Grand average ERP

The same ERP components that were examined in Experiment 1 were tested as a function of the cue type (2: uncued, cued), movement type (2: prosaccade, antisaccade), and the procedure type (2: blocked, mixed). There was a negative slow wave primarily in the central and parietal leads. A multivariate analysis of the FrontalZ, CentralZ, ParietalZ, and OccipitalZ electrode groups was tested with a Cue Type (2) × Movement Type (2) × Procedure Type (2) design. The effect of the cue type on the CentralZ virtual electrodes approached statistical significance, Wilk's Λ = 0.1860, *F*_(6, 5)_ = 3.65, *p* = 0.0885. As in Experiment 1, the negative slow wave was larger on the non-cued than on the cued trials. There was a significant interaction of the movement type and procedure type on the CentralZ virtual electrode group, Wilk's Λ = 0.1978, *F*_(12, 30)_ = 3.12. *p* = 0.0056. As in Experiment 1, there was no significant movement type effect on the CentralZ negative slow wave for the mixed-choice procedure. However, in the blocked trials, the negative slow wave was larger for the antisaccade trial block than for the prosaccade trial block (*M*'s of CentralZ virtual electrode group for prosaccade and antisaccade trial blocks = −4.06 and −4.60 μV, respectively).

The slow presaccadic positive slow wave occurring primarily over the parietal areas was examined by computing the mean value in the presaccadic interval from about −50 to −20 ms preceding the saccade (Figure 4, middle panel). This was analyzed for the FrontalZ, CentralZ, ParietalZ, and OccipitalZ electrode groups with a Cue Type (2) × Movement Type (2: prosaccade, antisaccade) × Procedure Type (2: blocked, mixed). There were no significant main effects or interactions involving the procedure type effect. This indicates that the positive slow wave occurring over the parietal leads was similar in magnitude for the blocked and mixed-choice trial types. Unlike this ERP component in Experiment 1, there was a main effect of the cue type on the ParietalZ electrode group, Wilk's Λ = 0.0731, *F*_(5, 6)_ = 15.21, *p* = 0.0024. The parietal slow wave was larger for the uncued trials than for the cued trials (*M*'s = 3.21 and 2.01 μV, respectively for uncued and cued trials). Even though the Cue Type × Procedure Type interaction was not significant, because this effect did not occur in Experiment 1 I tested the Cue Type effect for the blocked and mixed-choice procedure types separately with *post-hoc* error control methods. This cue type factor significantly affected the positive slow wave for the blocked trials (*p* < 0.05) but not for the mixed-choice trials.

The presaccadic spike potential was analyzed. The difference between the ERP in the intervals immediately preceding the saccade (−24 to −16 ms) and the ERP occurring at the time of the saccade (−8 through + 8 ms) was analyzed for the central electrode groups with a Cue Type (2) × Movement Type (2) × Procedure Type (2) MANOVA. There were no significant main effects or interactions involving the procedure type, indicating that the presaccadic spike potential was not significantly different for the blocked and mixed-choice trials. The effects found in Experiment 1 were also substantially replicated in Experiment 2, i.e., a significantly larger spike potential for the prosaccade eye movements in the CentralZ, ParietalZ, ipsilateral parietal electrode groups, than for the antisaccade eye movements in those electrodes.

#### ERP source analysis

There were two effects of the blocked trials on the ERP. First, on the blocked trials the negative slow wave was larger for antisaccade eye movements than for prosaccade eye movements. For the negative slow wave, the sources of the central negative slow wave was examined by computing the difference between the current density values from the last 50 ms of the pretarget interval and the first 50 ms, only for the blocked files. This was examined with a ROI (e.g., frontal pole, orbital frontal gyrus, Brodmann areas 6 and 8, dorsal ACC, ventral ACC, pre- and post-central gryi, superior parietal lobe, posterior cingulate, intraparietal sulcus, supramarginal gyrus, angular gyrus, dorsolateral PFC) × Movement Type (2) × Side (3: contralateral, central, ipsilateral) ANOVA. There were several main effects and interactions, including the three way interaction between ROI, movement type, and side, *F*_(9, 90)_ = 3.66, *p* = 0.0005. Three of the ROIs had a significant interaction between movement type and side. This occurred because the current density on the contralateral side was larger for antisaccade trials than prosaccade trials, but the current density on the ipsilateral side was not significantly different for prosaccade and antisaccade eye movements. This occurred for pre- and post-central gryi, the superior parietal lobe, and the frontal pole.

**Figure 9:**
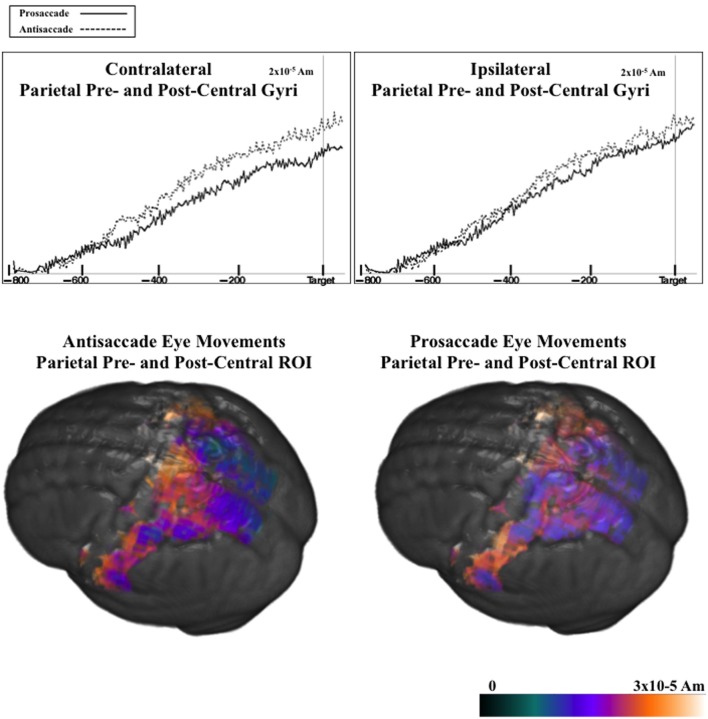
**(Top panel)** Temporal unfolding of the current density sources for the parietal, pre- and post-central gyri ROIs as a function of the eye movement type and side of the eye movement, for the pretarget ERP. **(Bottom panel)** Current density reconstruction for the pretarget ERP for the parietal, pre- and post-central ROIs, on prosaccade and antisaccade trials.

**Figure 10:**
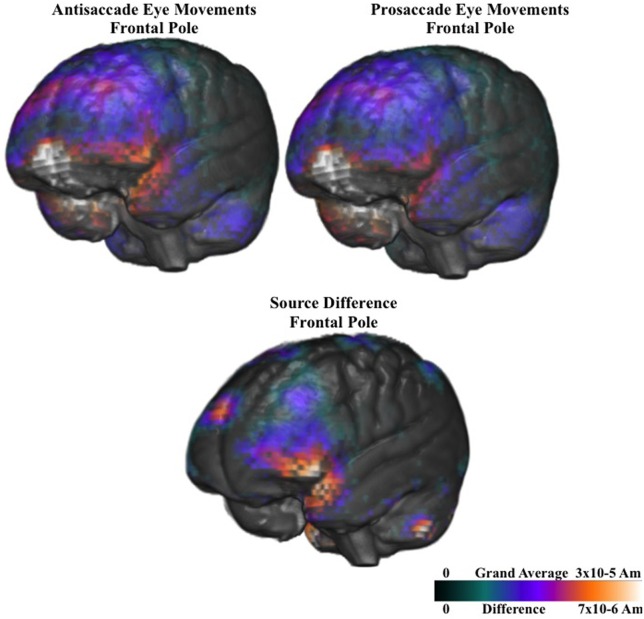
**Current density reconstruction of the pretarget ERP for the frontal pole activity for prosaccade and antisaccade eye movements**. The difference construction is for the difference between the prosaccade and antisaccade source models.

Figure 9 shows the ms-by-ms mean *nAm* for the parietal and pre- and post-central gyri ROIs combined, separate for the contralateral and ipsilateral sides and the prosaccade and antisaccade eye movements. The antisaccade trials resulted in larger current density the prosaccade trials in this parietal and mid-central areas on the contralateral side of the eye movement, but not on the ipsilateral side. Figure 9 also shows the parietal and pre- and post-central gyri ROI source activation for the prosaccade and antisaccade trials. The difference in the activity between the sides appeared in the most superior position of these ROI on the contralateral side of the eye movement. Figure 10 shows similar plots for the frontal pole area, for pro- and anti-saccade eye movements on the blocked trials. The activity was contralateral to the eye movement.

Second, there was a cue type effect for the presaccadic positive slow wave for the blocked trials that did not occur on the mixed-choice trials. The cortical sources of the presaccadic positive slow wave was examined by computing the difference between the current density values from presaccadic interval from about −50 to −20 ms preceding the saccade, only for the blocked trials. This was examined with an ROI × Cue Type × Movement Type ANOVA. There were some significant effects replicating the finding found in Experiment 1 for this ERP component. However, there were no significant effects or interactions involving the cue type effect or the movement type effect. This indicates that the current density for the cued and uncued trials was not significantly different in the blocked trials even though the ParietalZ component was affected by the trial type.

### Discussion

#### Pretarget and presaccadic ERP

There were three types of ERP activity found in this study that replicated findings from other studies. First, there was a negative potential shift in the ERP that occurred before target onset and was associated with the preparatory interval of the trial rather than the saccade itself. Several studies of ERP activity in the prosaccade and antisaccade eye movements report this negative shift in the EEG that begins up to 1 s prior to saccade onset and has its maximum over the vertex (Brickett et al., 1984; Evdokimidis et al., 1996; Everling et al., 1997, 1998; Klein et al., 2000; Richards, 2003; Mueller et al., 2009). This potential was similar to the “contingent negative variation” found in tasks with a preparatory interval and an imperative stimulus (e.g., S1-S2; CNV; Walter et al., 1964; Fabiani et al., 2000). In the current study using a block design, in which prosaccade and antisaccade trials are given in different blocks, this ERP component was larger in the antisaccade trial blocks than in the prosaccade trial blocks. In mixed-choice trials when the cue for the preparatory interval is informative for the type of eye movement, most often this negative presaccadic potential does not differ between prosaccade and antisaccade trials (Evdokimidis et al., 1996; Richards, 2003). The close link of this component to the preparatory period and its time course (500–1000 ms before saccade onset) suggests it represents the preparatory activity in mixed-choice trial designs, or response set in blocked designs, similar to the preparatory BOLD activity occurring in event-related fMRI studies. This presaccadic potential occurs for cued-catch trials (Richards, 2003) or in the current study when the cue was uninformative about the type of eye movement on the trial.

Two of the presaccadic activities were closely tied to events surrounding the saccade itself. The second type of ERP activity was the slow positive potential shift in ERP beginning about 100 ms before saccade onset with maximum values over central and parietal leads. This positive slow component over parietal leads is not unique to studies of the antisaccade and prosaccade but is also found in voluntary eye movements. Most studies report no difference in this component between antisaccade and prosaccade trials (Evdokimidis et al., 1996; Everling et al., 1997, 1998; Richards, 2003), but in the current study it was larger for antisaccade than for prosaccade trials in the mixed-choice procedure of Experiment 1. The third type of ERP component was the sharp spike in ERP over the central and parietal leads called the “spike potential.” In one study this potential was larger for antisaccade trials than prosaccade trials (blocked design, Everling et al., 1997) but generally this potential was the same on prosaccade and antisaccade trials (Evdokimidis et al., 1996; Everling et al., 1997, 1998; Klein et al., 2000; Richards, 2003). In the current study this ERP component was slightly larger on the prosaccade.

#### Cortical activation for saccade preparation

The cortical source analysis in the current study was useful in identifying the brain regions generating the ERP activity so it could be compared with neuroimaging studies using PET or fMRI. The studies of PET or fMRI using the block design find neural activity in this task in nearly every cortical area known to be involved in eye movement control in primates (FEF, SEF, superior parietal lobe, DPC [areas 9, 46], anterior cingulate cortex, anterior medial PFC [areas 8, 9], ventromedial PFC [area 10]; Everling and Fischer, 1998; Munoz and Everling, 2004; McDowell et al., 2008). Studies using event-related fMRI that separate the preparatory interval BOLD activity from response activity often report the BOLD activity in response to informative preparatory cues is larger on antisaccade than prosaccade trials in the FEF or SEF (SMA) (Curtis and D'Esposito, 2003, 2006; Desouza et al., 2003; Ford et al., 2005). The ERP activity in the current study similar to the preparatory BOLD response was the negative potential that began at the preparatory cue and preceded target onset. This ERP component was not different for prosaccade and antisaccade eye movements in the mixed-choice procedure used in Experiment 1. However, when the eye movement types were presented in separate testing blocks, the pretarget negative slow wave was larger for the antisaccade block. This effect is similar to that found with fMRI studies when the prosaccade and antisaccade trials are given in blocked trials or in mixed-choice trials. In fMRI studies there is an extended network of areas in the parietal and frontal lobes that are involved in the preparation of antisaccade eye movements (Ford et al., 2005; Brown et al., 2007; Ettinger et al., 2008). However, these areas appear to be linked to preparatory activity of the antisaccade and not saccade execution (e.g., Ford et al., 2005; anterior cingulate cortex, FEF, SEF, DPC, intraparietal sulcus, parietal-occipital sulcus). Many of these brain areas are enhanced for eye movements occurring in antisaccade trial blocks over prosaccade trials blocks, but are less widespread for mixed-choice trial blocks (Cornelissen et al., 2002; Dyckman et al., 2007).

The cortical sources of the pretarget negative slow wave were widespread in the current study, but the functional relation between eye movement type and these sources occurred primarily in the contralateral parietal and around the central gyrus (pre-central and post-central gyri). Studies using event-related fMRI that separate saccade preparatory activity from saccade execution have reported higher activation for antisaccade eye movements in similar areas (e.g., SMG in Ettinger et al., 2008; parietal-occipital sulcus in Ford et al., 2005). It should be noted that this ERP component did have cortical sources in portions of the anterior cingulate, especially the ventral regions. The sources in the anterior cingulate cortex were similar to several studies showing activity in blocked designs (Brown et al., 2004; Raemaekers et al., 2005; Matsuda et al., 2006) or event-related fMRI activity in the preparatory period (Ford et al., 2005; Brown et al., 2007). In addition to being larger for antisaccades than prosaccades, the area corresponding to the anterior portion of the cingulate gyrus shows a larger BOLD response in the preparatory interval on antisaccade trials that were correct compared to error trials (Ford et al., 2005). Both the anterior cingulate cortex and anterior portions of the cingulate gyrus have been shown to be active after saccades in this task differentially for error and correct antisaccades (Polli et al., 2005). This implies that this area is heavily involved in both saccade planning and eye movement evaluation in this task.

#### Cortical activation for saccade execution

The analysis of the responses immediately preceding the saccade and their cortical sources has implications for fMRI analysis. Both the presaccadic positive slow wave and the spike potential occurring at saccade onset appear to have their primary origin in the ventral areas of the prefrontal cortex. This includes the ventral region of the anterior cingulate cortex and the orbital frontal gyrus (Figure 6). The presaccadic positive slow wave was larger for antisaccade eye movements than for prosaccade eye movements, and this occurred on both the blocked and mixed-choice tasks. The close temporal relation of these components to the saccade onset is a finding uniquely suited for EEG/ERP work. These saccade-oriented effects cannot be shown in either blocked or event-related fMRI because of the temporal resolution of fMRI. It appears that the main effect of the blocking procedure vis-à-vis the eye movement types was on parietal and central brain areas rather than on prefrontal or anterior cingulate cortex. Alternatively, the responses specifically related to saccade execution occurred in the anterior cingulate and were not affected by the blocking manipulation. This finding suggests that the blocked trials used in typical fMRI (or ERP) studies results in a preparatory set that affects the brain areas controlling eye movement planning, whereas the brain areas more closely related to eye movement execution in the prefrontal cortex are unaffected by such preparatory sets.

One goal of this study was to improve the analysis of the cortical sources of the presaccadic ERP. The current study improved the analysis of the cortical sources of eye movement control over two prior studies (Richards, 2003; McDowell et al., 2005) in several ways. First, the McDowell et al. (2005) study used a “region-of-interest” approach and examined only a few selected cortical areas for the presaccadic ERP (lateral and medial FEF, SEF, and DPC). The extensive involvement in the current study of the anterior cingulate cortex, frontal pole, and orbital-frontal areas in the brain activity immediately preceding the eye movement therefore would have been overlooked. Second, these prior studies used a single MRI for the identification of source locations (average of individual MRIs in McDowell et al., 2005; single MRI in Richards, 2003) and McDowell et al. averaged the source locations to show continuous source areas. The use of an averaged MRI, generic brain, or normalized space leads to an artificial restriction of source locations and apparent localization precision, whereas the averaging of different underlying scalp sources may lead to smearing of the EEG (ERP) potential and smearing of the source locations. Doing source analysis tailored to the individual's anatomical space may be particularly important in the presence of significant differences in head size or the relation of the brain areas to scalp landmarks (Ha et al., 2003). Third, the use of finite element methods (FEM) for the resistance pathways for calculating the forward model (Rosenfeld et al., 1996; Awada et al., 1997; Buchner et al., 1997; Michel et al., 2004; Slotnick, 2004) was an improvement over the three-shell spherical model used by Richards (2003) or the three-compartment boundary element model used by McDowell et al. (2005). The boundary element models are geometrically more realistic than the spherical models, but cannot faithfully represent changes in resistance between gray matter, white matter, CSF, and muscle within the central compartment. The FEM models account for non-homogenous tissue within the head, local variations in tissue depth or area, and electrical anisotropies; though in practice, boundary element models and finite element models may give very similar results (Slotnick, 2004).

## Summary: comparing fMRI and ERP for studying eye movements

The results of the cortical analysis in the current study compare favorably with neuroimaging studies using PET, block-design fMRI, and event-related fMRI. This study and others (Evdokimidis et al., 1996; Everling et al., 1998; Richards, 2003) showed the negative presaccadic potential was related to preparatory responses to the target and not the events surrounding the saccadic eye movements. Several studies using event-related fMRI showed that activity in the areas of the brain that differentiate antisaccade from prosaccade eye movements (e.g., FEF) have substantial preparatory BOLD activity (Cornelissen et al., 2002; Curtis and D'Esposito, 2003; Desouza et al., 2003; Ford et al., 2005; Ettinger et al., 2008). In these studies when the BOLD activity was linked to the saccade execution period, some of the differentiation for antisaccades and prosaccades was eliminated. In addition to the likelihood that blocked presentations lead to preparatory set enhancement of the antisaccade brain activity, it also may be the case that in mixed-choice trials with informative cues indicating the upcoming eye movement that antisaccade eye movements are preceded by more activity than prosaccade eye movements (e.g., Richards, 2003; Brown et al., 2007, and Experiment 1 cueing effects). Both blocked presentations and instructional cues lead to shorter reaction times at the target onset, and a smaller difference between antisaccade and prosaccade trials (Richards, 2003; and both experiments of this study). Although the event-related fMRI design can separate blocked trials and mixed-choice trials effects and can separate preparatory effects from eye movement execution effects, the temporal resolution of the fMRI neuroimaging technique cannot identify the temporal process of brain activity surrounding the saccade (c.f. 4 s preparatory effects with 0.5 s saccade effects, Figure 1 in Ford et al., 2005). Studies using ERP and cortical source analysis (e.g., current study; Richards, 2003; McDowell et al., 2005) identified brain areas that were associated with the saccadic eye movement and pinpointed brain activity in the 100 ms preceding the saccade. I conclude that ERPs with cortical source analysis are useful in differentiating brain activities associated with general preparatory processes for control and brain activities associated with eye movement execution.

### Conflict of interest statement

The author declares that the research was conducted in the absence of any commercial or financial relationships that could be construed as a potential conflict of interest.

## Footnotes

^1^The choice of 100 kW as the maximum impedance value was based on the high input impedance of the EGI amplifiers. These amplifiers have an input impedance of about 200 MW compared with traditional EEG amplifier impedances of about 10 MW. Given the recommendation of interelectrode impedances being at least 1% of amplifier input impedance (e.g., 10 kW for 10 MW amplifier; Picton et al., 2000), 100 kW is appropriate for this amplifier. Ferree et al. (2001) estimate that for this amplifier system a 50 kW preparation would lead to a maximum 0.025% signal loss, and therefore the current levels should lead to no more than a 0.050% signal loss. They found no discernible signal loss with electrode preparations at about 40 kW.

^2^The ANOVAs for the analyses were done with a general linear models approach using non-orthogonal design because of the unequal number of eye movements across factors, and because of the different numbers of eye movements across subjects (see Searle, 1971, 1987; Hocking, 1985). In all analyses, the Scheffe method was used to control for inflation of testwise error rate for post hoc comparisons. The error mean squares for each post-hoc comparison was obtained from the error term for the omnibus interaction for that post-hoc evaluation. The significance of the post-hoc tests was *p* < 0.05 for all tests and these individual probabilities were not reported in the text.
