# Supplementary material for: Cortical sources of ERP in prosaccade and antisaccade eye movements using realistic source models
Source: Front Syst Neurosci. 2013 Jul 2;7:27. doi: 10.3389/fnsys.2013.00027 (PMC3698448; doi:10.3389/fnsys.2013.00027)
Supplement: Supplementary file 1 [file DataSheet1.DOCX]

Supplemental Information for “Cortical Sources of ERP in Prosaccade and Antisaccade Eye Movements using Realistic Source Models ”

**Virtual 10-20 Electrodes**

The GSN 128 Sensornet channel electrodes were combined into groups of electrodes representing “virtual 10-20” electrodes. For ERP grand average analyses the electrodes were grouped into sets of electrodes that were close in distance to the 10-20 electrode configuration. Supplemental Figure 1 shows the GSN sensor net with the electrodes near the 10-20 (+ Oz) electrodes, and with virtual 10-20 electrodes marked. A list of the electrode locations and the virtual 10-20 electrodes is given in a Table 1.

**Head models for individual participants.**

The models used structural MRIs from individual participants to restrict the source solution to the gray matter of that participant. This allowed the source locations to be restricted to gray matter locations for that participant and defined specific anatomical areas tailored to the individuals’ anatomical space rather than a generic brain or normalized Talairach space (Ha, Youn, Kong, Park, Ha, Kim, & Kwon, 2003). A “region-of-interest” (ROI) approach was used, where anatomical ROIs were defined by anatomical stereotaxic atlases based on the individual participants’ MRI. The MRIs were also segmented into media (gray matter, white matter, CSF, skin, skull, muscle, eye, nasal cavity) and realistic resistance models were used to calculate the forward model with a finite element method (FEM) for the resistance pathways (Awada, Jackson, Williams, Wilton, Baumann, & Papanicolaou, 1997; Buchner, Knoll, Fuchs, Rienacker, Beckmann, Wagner, Silny, & Pesch, 1997; Michel, Murray, Lantz, Gonzalez, Spinelli, & Grave de Peralta, 2004; Rosenfeld, Tanami, & Abboud, 1996; Slotnick, 2004). The realistic models should improve the accuracy of the source localization while retaining the temporal advantage that ERP has over MRI- or PET-based neuroimaging methods.

**Electrode Locations on MRI**

The MRIcron program (Rorden, <http://www.mccauslandcenter.sc.edu/mricro/mricron/>) was used to display MRIs and do editing work. The anterior commisure and posterior commisure were located in the MRI. The anterior commisure was defined as the origin, the line between the anterior and posterior commisure as the coronal axis, and the perpendicular lines bisecting the anterior commisure as the coronal and saggital planes (i.e., Talairach coordinate system; Talairach & Tournoux, 1988). The MRI was used to identify a number of “fiducial locations” on the skull (nasion, inion, mastoids, preauricular skull locations).

The GSN 128 channel electrode locations were estimated on each individual participant MRI. Each participant had a GSN 128 channel Sensor Net (GSN128) placed on their head during the recording. Photographs of the net placements were taken from the front, rear, right, left, and above the head. The photos were used to visually identify the position of electrodes on the participant MRI volume on the front, rear, left and right of the head (GSN #’s 17, 73, 57, 101, respectively) and the electrode in the Cz location (average of GSN electrodes 7, 31, 55, 80, 106). These electrodes were visually located on the scalp of the participant’s MRI volume and translated into the AC-PC space of that individual. These electrode points were registered to an average GSN adult electrode configuration (Richards et al., 2013) using “coherent point drift” registration (CPD version 2; Myronenoko et al, 2006; Myronenko & Song, 2010). The resulting 12 degree of freedom affine registration matrix was used to transform the average electrode configuration into the participant space. This transformed electrode configuration was then fitted to the analogous electrode points on the individual MRI volume, and each electrode was fitted to the scalp by finding the nearest location to the scalp from the electrode. The resulting electrode locations were referenced to the AC-coordinate system for that participant.

**Head Segmentation**

The materials in the head were segmented, including scalp, skull, CSF, white matter, gray matter, nasal cavity, and eyes (Richards, 2002, 2005). The FSL computer program (Smith et al., 1999) was used for the brain extraction (BET2, Smith, 2002), the identification of the skull and scalp (BETSURF, Jenkinson, Pechaud, & Smith, 2005), and the segmentation of the brain into WM, GM, and other (FAST; Zhang, Brady, & Smith, 2001). Three-dimensional tetrahedral wireframes were computed that contained the location of each corner of the tetrahedron and the type of material making up the tetrahedron, using the MR Viewer module of the EMSE computer program (Source Signal, Inc). These wireframe files had tetrahedral voxel sizes of 2 mm-cubed, with from 55K to 90K vertices, and 260K to 450K tetrahedra for each wireframe. The FEM wireframe generated by the MR Viewer program was used with a specialized computer program to assign conductivity values to each tetrahedral segment proportional to the amount of segmented material in the tetrahedron. Figure 1 (top row) shows the segmented wireframe from an anatomical MRI from one participant. The MRIs were displayed with the MR Viewer program or the MRICron program

**Atlases**

Five stereotaxic atlases were constructed for each participant MRI. First, three atlases were constructed on a 20-24 year old MRI template (Phillips et al., 2013). A manually drawn atlas was constructed on each average MRI template. This was done by manual segmentation of the cerebral lobes of the brain (frontal, temporal, parietal, occipital, insular), sub-lobar cerebral areas (cingulate cortex, fusiform gyrus), sub-cortical (striatum, thalamus, corpus callosum) and non-cortical areas (brainstem, cerebellum, and ventricles) (Philips et al., 2013). For the atlases from the template, the participant’s brain was extracted from the T1W MRI and registered with linear affine methods to the average template using the FSL FLIRT program (Jenkinson & Smith, 2001). The affine matrix was used to inverse transform the atlas in the template space to the participant space. Thus each individual had a regional stereotaxic atlas derived from the average MRI template atlas. The 20-24 year old template also had a Harvard-Oxford (Desikan, Segonne, Fischl, Quinn, Dickerson, Maguire, Hyman, Albert, Killiany, 2006) atlas and Brodmann atlas obtained from the FSL computer program. These atlases were transformed with the flirt affine matrix to the head space of the individual participant.

Two atlases were constructed on the individual participants MRIs. The LONI Probabilistic Brain Atlas (LPBA; Shattuck, et al., 2008) and the Hammers atlas, based on MRIs from the Information Exchange for the Internet (Hamers atlas; Heckemann, Hajnal, Aljabar, Rueckert, & Hammers, 2006; Heckemann, et al., 2003) were constructed on individual participants. The atlases for individual participants were done using methods described by Gousious et al. (2008). The LPBA atlas was developed by manual segmentation of 40 individual adult MRIs from the LONI MRI database (Shattuck, et al., 2008). This segmentation was done in 56 areas for the cortex, subcortex, brainstem and cerebellum. For each individual MRI, the extracted brain was linearly registered to the 40 adult brains and each segmented adult atlas was transformed to the participant space. The resulting atlases were fused in a majority vote procedure (Gousias, et al., 2008; Shi, et al., 2011). The majority vote fusions was done by aggregating the different segments across the 30 individuals, and assigning to each voxel the segment number which had the highest number of modal votes (highest partial volume estimate) for that voxel. If two segments had the same number of votes for a voxel (equal PVE value), then a MRI volume with randomly assigned volumes was used to mask the voxels, with a result that one or the other segment was randomly chosen for that voxel. These competitive decisions occurred at the borders of segments and were less than 5% of the volume voxels. The resulting atlas identifies the 56 brain locations for each voxel of the individual participant brain. The same procedure was done for the Hammers segmented areas. The Hammers atlas was developed by manual segmentation of 30 individual adult MRIs from the IXI MRI database (Heckemann, et al., 2003) and identified 83 areas from the cortex, subcortex, brainstem and cerebellum. The same procedure used to construct the LPBA atlas was done with the Hammers atlases, resulting in a Hammers atlas for each individual. Table 1 of the Supplemental Information has a list of the regions-of-interest that were used in the current study, and the segment areas from the Harvard-Oxford, Brodmann, LPBA, and Hammers atlases. Details of these procedures can be found in Phillips et al. (2012).

The atlases were used to identify “Regions of Interest” (ROIs) in the participant brain. This was done by using the appropriate atlas designation for anatomical areas that were hypothesized to be related to the antisaccade or prosaccade eye movements, and to the effects of the spatial cueing on the eye movement related brain activity. The areas were defined by combining the appropriate masks from each atlas for a single area. Bilateral (non-lateralized) volumes were defined for the frontal pole, orbito-frontal cortex, ventral anterior cingulate, dorsal anterior cingulate, posterior cingulate, and superior parietal lobe. Lateral (separate right and left) volumes were defined for the combined Brodmann areas 6 and 8, frontal pole, superior parietal lobe, and an area including both the precentral and postcentral gyri. Figure 1 (middle and bottom panels) shows the ROI source volumes on an individual participant for the frontal pole, orbito-frontal, Brodman areas 6 and 8, and cingulate gyrus. The Supplemental Infomration figure shows these ROI source volumes on a 3-D rendered brain.
